# Mixed supplementation of dietary inorganic and organic selenium modulated systemic health parameters and fecal microbiota in weaned pigs

**DOI:** 10.3389/fvets.2025.1531336

**Published:** 2025-02-14

**Authors:** Hyunjin Kyoung, Ikcheol Shin, Younghoon Kim, Jin Ho Cho, Kyeong Il Park, Yonghee Kim, Jinmu Ahn, Jinuk Nam, Kimoon Kim, Yonggu Kang, Hyeun Bum Kim, Minho Song

**Affiliations:** ^1^Division of Animal and Dairy Science, Chungnam National University, Daejeon, Republic of Korea; ^2^Department of Agricultural Biotechnology and Research Institute of Agriculture and Life Science, Seoul National University, Seoul, Republic of Korea; ^3^Department of Animal Science, Chungbuk National University, Cheongju, Republic of Korea; ^4^Department of Animal Resources Science, Dankook University, Cheonan, Republic of Korea

**Keywords:** blood biochemical parameters, fecal microbiota, immune responses, selenium, weaned pigs

## Abstract

This study was conducted to evaluate the effects of dietary mixed selenium [MSe: inorganic selenium (ISe) + organic selenium (OSe)] levels on the growth performance, blood parameters, and fecal microbiota of weaned pigs. In a randomized complete block design (block = initial body weight), 156 weaned pigs were allotted to three dietary treatments (4 pigs per pen; 13 replicates per treatment) for 42 days. Dietary treatments included (1) a non-Se-fortified diet based on corn and soybean meal (CON), (2) CON + 0.15 ppm ISe and 0.15 ppm OSe (MSe3), and (3) CON + 0.25 ppm ISe and 0.25 ppm OSe (MSe5). Pigs fed both MSe diets showed no effects on growth performance or diarrhea frequency compared with those fed the CON diet. However, pigs fed MSe3 and MSe5 had higher serum interleukin-6 (*p* = 0.021, linear *p* = 0.011) on day 7 and higher Se concentrations (*p* = 0.002, linear *p* = 0.001) on day 42 than those fed the CON. In addition, pigs fed different levels of MSe exhibited quadratic (*p* = 0.054) and linear (*p* = 0.069) effects on the number of white blood cells and hematocrit on day 42 compared with those fed CON, respectively. Moreover, the MSe3 group had higher total protein concentration (*p* = 0.049, quadratic *p* = 0.026) on day 42 than the CON group, and the MSe5 group had lower blood urea nitrogen concentration (*p* = 0.094, linear *p* = 0.033). There were no differences in alpha diversity indices of fecal microbiota among dietary treatments. However, beta diversity indices based on the Bray–Curtis dissimilarity were clustered differently (*r*^2^ = 0.56, *p* = 0.001) among dietary treatments. Pigs fed the MSe5 diet showed an increase in the relative abundance of phylum Bacteroidetes [false discovery rate (FDR) adjusted *p* = 0.004], families Barnesiellaceae (FDR adjusted *p* = 0.006) and Veillonellaceae (FDR adjusted *p* = 0.006), genera *Barnesiella* (FDR adjusted *p* = 0.023) and *Megasphaera* (FDR adjusted *p* = 0.023), and species *Barnesiella intestinihominis* (FDR adjusted *p* = 0.016) and *Megasphaera elsdenii* (FDR adjusted *p* = 0.019) compared with those fed the CON diet. In conclusion, dietary MSe modulated the systemic health parameters and fecal microbial community in weaned pigs.

## Introduction

Dietary selenium (Se), as an essential nutrient trace mineral, plays an important role in biological functions and is vital in animal feed. However, insufficient nutritional Se in swine feed can lead to white muscle disease and mulberry heart disease, while excessive supplementation can lead to anemia, hair loss, and organ damage (1, 2). In addition, excessive Se excretion can be linked to ecological pollution (3), which should be considered for sustainable swine production. Thus, the dietary Se content in swine feed was suggested by the National Research Council and Food and Drug Administration to be 0.15 to 0.30 ppm and not greater than 0.30 ppm, respectively, considering the requirements of pigs (4, 5). Dietary Se is noted for its antioxidant properties along with vitamin E. The diverse physiological effects of Se, including antioxidation and redox regulation against reactive oxygen species (ROS), are mediated by selenoproteins (SePs) and also play important roles in thyroid hormone metabolism and reproduction and muscle function (6, 7). Previous studies have shown that supplementation of dietary Se improves the immune system and reduces intestinal inflammatory responses due to its antioxidant effects, which are beneficial for the growth and health of pigs (8, 9). Therefore, to prevent Se deficiency or selenosis and achieve optimal growth and health of pigs, additional dietary Se supplementation to their feed beyond that obtained from feed ingredients and feeding in adequate amounts are necessary.

Dietary Se, provided by supplementing it to animal feed according to their nutritional requirements, is in inorganic (ISe) and organic (OSe) forms. Ingested dietary ISe or OSe is absorbed in the small intestine and transported (ISe, passive transport via the diffusion process; OSe, active transport via amino acid pathway) to the liver through the bloodstream and then redistributed to other tissues in the body after producing SePs in the liver (4, 10). Finally, Se is excreted in feces (mainly OSe) or urine (mainly ISe) through filtration and utilization of Se to protect the cell membranes in performing kidney functions (2, 11). Differences in the metabolic pathways in the body depending on the Se form result in different bioavailability and biological effects. Swine nutritionists have conducted studies comparing the effects of different sources and levels of dietary Se. At selenosis levels (over 5 ppm), dietary ISe caused more severe and rapid clinical signs than dietary OSe, with OSe inducing less severe effects than ISe as the addition level increased (1). In addition, dietary OSe was more efficient than dietary ISe in utilization and retention in tissues and blood (1, 2, 12). However, economic factors are important in swine production, and dietary ISe is widely used in pig feed because it is more efficient in supply and cost than dietary OSe (13). Interestingly, mixed Se (MSe: ISe + OSe) feeding in poultry has been suggested to improve production performance and health compared with individual Se feeding (14, 15). However, research on the effects of dietary MSe on the growth and health of pigs is unclear. Moreover, although tissues and blood indicators that reflect the Se status of the body are well known (2, 11), the effects of dietary Se on the gut microbiota and microbial indices in pigs are limited. Although weaned pigs are prone to gut microbial imbalance due to weaning stress, dietary Se supplementation can enhance gut health by promoting the growth of beneficial bacteria such as *Lactobacillus* and suppressing potentially harmful bacteria such as *Escherichia coli* (16, 17). These effects indicate that supplementing the weaner diet with MSe may help modulate gut microbiota, supporting the overall health of pigs. Therefore, this study aimed to evaluate the effects of different levels of dietary MSe on growth performance, diarrhea frequency, blood parameters, and fecal microbiota of weaned pigs. In addition, to support the effects of different levels of dietary MSe on weaned pigs, we set the maximum supplemental MSe level at 0.5 ppm to minimize adverse effects while providing effective recommendations.

## Materials and methods

### Animal ethics

The protocol for this study was reviewed and approved by the Institutional Animal Care and Use Committee of Chungnam National University, Daejeon, South Korea (approval #: 202012A-CNU-168).

### Experimental design, animals, and diets

In a randomized complete block design [block: initial body weight (BW)], newly weaned pigs [*n* = 156; (Landrace × Yorkshire) × Duroc; initial BW = 7.85 ± 1.11 kg; 28 days old] were randomly allotted to one of three dietary treatments (4 pigs per pen; 13 replicate pens per treatment). Dietary treatments were (1) a non-Se-fortified diet based on corn and soybean meal (CON), (2) CON +0.15 ppm ISe and 0.15 ppm OSe (MSe3), and (3) CON +0.25 ppm ISe and 0.25 ppm OSe (MSe5). The non-Se-fortified diet was formulated according to the nutritional requirements of weaned pigs (5), except for Se (Table 1). The Se products were obtained from commercial suppliers (ISe, sodium selenite, 1,000 ppm, Daone Chemical Co., Ltd., South Korea; OSe, Se-yeast, 1,000 ppm, Alltech Korea, Co., Ltd., South Korea). To determine the Se concentration in the feed, all diet samples were digested in a digestion block (N-Biotek, South Korea), allowed to react with 2,3-diaminonaphthalene solution, and then analyzed using the fluorometric method (18) with a fluorescence spectrometer (RF-6000, Shimadzu Co., Kyoto, Japan), as reported by the Association of Official Agricultural Chemists (AOAC) (method 996.16) (19). The analyzed Se contents in the CON, MSe3, and MSe5 groups were 0.086, 0.261, and 0.456 ppm, respectively. The pigs had free access to mash feed and water during the 42-day experimental period. The pigs were housed in pens of the same size, and ambient temperature (25–28°C), humidity (50–60%), and lighting (12-h intervals) were automatically controlled.

**Table 1:** Composition of experimental diets for weaned pigs (as-fed basis).

Item	Basal diet
Ingredient, %	
Corn	55.11
Soybean meal, 44%	37.00
Beef tallow	2.50
Meat and bone meal	2.00
Mono-dicalcium phosphate	0.95
Limestone	0.92
Vitamin–mineral premix^1^	0.20
l-lysine-HCl	0.84
dl-methionine	0.29
l-threonine	0.19
Total	100.00
Calculated energy and nutrient content	
Metabolizable energy, kcal/kg	3,400
Crude protein, %	24.30
Calcium, %	0.85
Phosphorus, %	0.70
Lysine, %	1.70

^1Provided per kilogram of diet: vitamin A, 12,000 IU; vitamin D3, 2,500 IU; vitamin E, 30 IU; vitamin K3, 3 mg; d-pantothenic acid, 15 mg; nicotinic acid, 40 mg; choline, 400 mg; and vitamin B12, 12 μg; Fe, 90 mg from iron sulfate; Cu, 8.8 mg from copper sulfate; Zn, 100 mg from zinc oxide; Mn, 54 mg from manganese oxide; I, 0.35 mg from potassium iodide.^

### Data and sample collection

The individual BW of pigs and feed residual in the feeder were estimated at the end of the study to measure the average daily gain, average daily feed intake, and gain to feed ratio on a pen basis. The fecal score was checked daily for the first 2 weeks with a score of 1 to 5 (1 = normal feces, 2 = moist feces, 3 = mild diarrhea, 4 = severe diarrhea, and 5 = watery diarrhea) by 2 independent researchers (20). The diarrhea frequency was calculated by counting pen days with a pen diarrhea score of 4 or higher. Blood samples were collected from one pig per pen randomly selected from six replicate pens using 10 mL tubes (BD Vacutainer Systems, Franklin Lakes, NJ, United States) with or without ethylenediaminetetraacetic acid (EDTA) (21) via the jugular vein of the pigs on days 7, 14, and 42. The collected blood samples in non-EDTA tubes were centrifuged at 3,000 rpm for 15 min at 4°C to obtain serum samples, which were then stored at −80°C for subsequent blood analysis. On the last day of the study, fecal samples were collected from one pig per pen randomly selected from six replicate pens using 15 mL conical tubes via rectal stimulation with a sterile swab and stored at −80°C for microbiota analysis (22).

### Blood analyses

The blood samples collected in the EDTA tubes were analyzed for the number of white blood cells, red blood cells, hemoglobin, hematocrit, platelet, mean corpuscular volume, mean corpuscular hemoglobin, and mean corpuscular hemoglobin concentration using an automated hematology analyzer (scil Vet abc hematology analyzer, scil Animal Care Company, Altorf, France). Serum samples for Se concentration analysis were digested in a digestion block (N-Biotek, South Korea), allowed to react with 2,3-diaminonaphthalene solution, and analyzed using a fluorescence spectrometer (RF-6000, Shimadzu Co., Kyoto, Japan) based on the fluorometric method (23) according to the AOAC (19). Other serum samples were thawed at room temperature and analyzed for immune responses (cortisol, tumor necrosis factor-*α*, transforming growth factor-β1, interleukin-1β, and interleukin-6) and biochemical parameters [total protein, calcium, inorganic phosphorus, magnesium, total cholesterol, triglyceride, glucose, albumin, creatinine, blood urea nitrogen (BUN), aspartate aminotransferase (AST), alanine aminotransferase (ALT), and AST to ALT ratio (AST:ALT)] using enzyme-linked immunosorbent assay kits (R&D System Inc., Minneapolis, MN, United States) with a microplate reader (Epoch microplate spectrophotometer, BioTek Instruments Inc., Winooski, VT, United States) (24) and a clinical autoanalyzer (Toshiba Acute Biochemical Analyzer-TBA-40FR, Toshiba Medical Instruments, Tokyo, Japan) with a specific kit (Wako Pure Chemical Industries, Osaka, Japan) (25), respectively. All serum analyses were performed in duplicate using appropriate blood volumes according to the manufacturer’s instructions.

### Fecal microbiota analysis

Fecal samples were analyzed at a biotechnology company (Macrogen Inc., Seoul, South Korea). After extraction of the total DNA from 200 mg of fecal samples using a QIAamp Fast DNA Stool Mini Kit (QIAGEN, Hilden, Germany), DNA concentration and purity were checked using a NanoDrop ND-1000 spectrophotometer (NanoDrop Technologies, DE, United States). The V3–V4 regions of the 16S rRNA gene were amplified using the Bakt 341F-805R primers to construct an amplicon library. Pair-end sequences using the MiSeq platform (Illumina, San Diego, CA, United States) were merged using FLASH v. 1.2.11. To perform preprocessing and clustering, the CD-HIT-OTU program of CD-HIT v. 4.5.4 was used to eliminate sequencing errors by identifying and removing low-quality reads, ambiguous reads, and chimeric reads. The remaining reads then clustered into OTUs at a 97% identify cutoff (26). Taxonomic assignment was performed using BLAST+ v. 2.9.0 with reference to the NCBI 16S microbial database. QIIME was performed to analyze gut microbial community comparisons based on OTU abundance and taxonomy information. Microbial alpha diversity indices (observed OTUs, Chao1, Shannon, and Simpson) and beta diversity indices [principal coordinate analysis (PCoA) plots based on the Bray–Curtis dissimilarity] were measured for richness and evenness within samples and differences in the community among samples, respectively. Microbial data were normalized by data scaling using the total sum scaling before statistical comparison.

### Statistical analyses

Data were analyzed using the MIXED procedure of SAS software (v. 9.4; SAS Institute Inc., Cary, NC, United States) with a randomized complete block design (block: initial BW). The experimental unit was the pen. The statistical model for growth performance, blood profiles, immune responses, and blood biochemical parameters included dietary treatments as the main effect and block as a random effect. Treatment means were calculated using the LSMEANS statement, and means were separated using the PDIFF option in the PROC MIXED. Contrast statements were used to evaluate the linear and quadratic effects of dietary MSe levels, with PROC IML of SAS generating coefficients for unequally spaced levels. Diarrhea frequency was analyzed using the chi-square test of SAS. The MicrobiomeAnalyst webtool^1^ was used for analyzing fecal microbiota diversities (alpha diversity, the Kruskal–Wallis test; beta diversity, permutational multivariate analysis of variance) and comparison and classification [linear discriminant analysis (LDA) effect size (LEfSe), LDA score ≥ 2.0, false discovery rate (FDR) adjusted *p*-value <0.05]. Pearson correlation between featured microbial species through LEfSe analysis and blood biochemical parameters were analyzed using SAS. The results are presented as means ± standard error of the mean. Statistical significance and tendency among dietary treatments were considered at *p* < 0.05 and 0.05 ≤ *p* < 0.10, respectively.

## Results

### Growth performance and blood selenium level

There were no differences in the average daily gain, average daily feed intake, and gain to feed ratio during the experimental period among the CON, MSe3, and MSe5 groups (Table 2). Additionally, diarrhea frequency during the first 2 weeks after weaning was not affected by dietary MSe in addition to weaner feed. However, pigs fed dietary MSe3 and MSe5 had higher (*p* = 0.002, linear, *p* = 0.001) serum Se concentration on day 42 than those fed CON.

**Table 2:** Effects of dietary mixed selenium on overall growth performance and blood selenium concentration of weaned pigs^1^.

	Dietary treatments				*p*-value		
Item^2^	CON	MSe3	MSe5	SEM	Diet	Linear	Quadratic
Growth performance (day 1–42)							
Initial body weight, kg	7.85	7.86	7.85	0.32	0.999	0.993	0.972
Final body weight, kg	22.49	22.29	22.15	0.84	0.958	0.771	0.999
Average daily gain, g/d	348.75	343.54	340.51	15.45	0.930	0.705	0.989
Average daily feed intake, g/d	641.15	649.56	644.37	22.59	0.965	0.899	0.817
Gain to feed ratio, g/g	0.546	0.528	0.531	0.019	0.774	0.540	0.717
Frequency of diarrhea, %	10.99	14.29	14.84		0.592		
Selenium concentration (day 42)							
Serum, ppm	0.088^b^	0.147^a^	0.154^a^	0.012	0.002	0.001	0.210

^1^Each value for growth performance and selenium concentration are the mean of 13 replicates (4 pigs per pen) and 6 replicates (1 pig per pen), respectively.

^2^CON, a non-selenium-fortified diet based on corn and soybean meal; MSe3, CON + 0.15 ppm inorganic selenium + 0.15 ppm organic selenium; MSe5, CON + 0.25 ppm inorganic selenium + 0.25 ppm organic selenium; Diet, statistical comparisons among dietary treatments; Linear, linear contrast of dietary mixed selenium supplementation levels; Quadratic, quadratic contrast of dietary mixed selenium supplementation levels. Frequency of diarrhea for the first 2 weeks after weaning = (number of diarrhea score of 4 or higher/number of pen days) × 100.

^a,b^Means with different superscript letters within each variable are different (*p* < 0.05).

### Hematological, immunological, and biochemical parameters

Supplementation of different levels of dietary MSe to a weaner diet had no effect on blood profiles on day 7 (Table 3). However, mean corpuscular hemoglobin concentration (MCHC) showed a tendency for a quadratic contrast (*p* = 0.059) on day 14 following dietary MSe addition. The number of white blood cells and hematocrit exhibited tendencies for quadratic (*p* = 0.054) and linear (*p* = 0.069) contrasts on day 42 among the dietary treatments, respectively. Additionally, hemoglobin level had a linear contrast (*p* = 0.048) on day 42 following increased MSe levels. Moreover, pigs fed MSe3 tended to have lower (*p* = 0.058, quadratic *p* = 0.039) mean corpuscular hemoglobin (MCH) on day 42 than those fed CON. As shown in Table 4, supplementation of dietary MSe3 and MSe5 had higher (*p* = 0.021, linear *p* = 0.011) serum interleukin-6 concentration on day 7 than the CON but did not differ from each other. In addition, there were no differences in serum immune responses on day 14 following the addition of dietary MSe. The results presented in Table 5 show that dietary MSe influenced the blood biochemical parameters in weaned pigs. Pigs fed MSe3 had higher (*p* = 0.049, quadratic *p* = 0.026) total protein level on day 42 than those fed CON but did not differ from those fed MSe5. In addition, dietary MSe5 tended to lower (*p* = 0.094, linear *p* = 0.033) BUN level on day 42 than the CON.

**Table 3:** Effects of dietary mixed selenium on blood profiles of weaned pigs^1^.

	Dietary treatments				*p*-value		
Item^2^	CON	MSe3	MSe5	SEM	Diet	Linear	Quadratic
White blood cells, ×10^3^/μL							
Day 7	21.83	21.92	17.13	2.67	0.375	0.268	0.392
Day 14	26.93	24.87	26.20	2.97	0.884	0.823	0.663
Day 42	21.12	30.18	18.95	4.02	0.145	0.889	0.054
Red blood cells, ×10^6^/μL							
Day 7	7.37	7.78	7.37	0.23	0.372	0.854	0.170
Day 14	8.05	7.71	7.79	0.30	0.711	0.514	0.623
Day 42	6.88	6.79	6.33	0.30	0.399	0.236	0.523
Hemoglobin, g/dL							
Day 7	12.65	13.07	12.58	0.42	0.690	0.991	0.397
Day 14	13.77	12.75	13.33	0.44	0.286	0.399	0.181
Day 42	12.48	11.62	11.22	0.42	0.130	0.048	0.841
Hematocrit, %							
Day 7	38.67	40.17	38.27	1.39	0.603	0.931	0.324
Day 14	41.40	39.18	39.48	1.52	0.550	0.352	0.579
Day 42	30.27	28.68	27.30	1.07	0.180	0.069	0.884
Platelet, ×10^3^/μL							
Day 7	492.50	480.83	364.17	74.70	0.426	0.272	0.489
Day 14	487.00	359.50	423.00	60.96	0.360	0.391	0.254
Day 42	462.67	479.17	454.17	33.77	0.869	0.907	0.612
Mean corpuscular volume, fL							
Day 7	52.67	51.67	51.83	0.77	0.626	0.420	0.607
Day 14	51.50	50.83	50.50	0.80	0.675	0.385	0.947
Day 42	44.17	42.33	43.17	0.59	0.120	0.179	0.109
Mean corpuscular hemoglobin, pg							
Day 7	17.20	16.78	17.10	0.29	0.571	0.719	0.326
Day 14	17.10	16.62	17.15	0.27	0.341	0.964	0.149
Day 42	18.17	17.20	17.77	0.26	0.058	0.196	0.039
Mean corpuscular hemoglobin concentration, g/dL							
Day 7	32.80	32.47	33.00	0.28	0.428	0.730	0.215
Day 14	33.25	32.68	33.80	0.36	0.119	0.401	0.059
Day 42	41.22	40.53	41.12	0.37	0.393	0.731	0.192

^1^Each value is the mean of 6 replicates (1 pig per pen).

^2^CON, a non-selenium-fortified diet based on corn and soybean meal; MSe3, CON + 0.15 ppm inorganic selenium + 0.15 ppm organic selenium; MSe5, CON + 0.25 ppm inorganic selenium + 0.25 ppm organic selenium; Diet, statistical comparisons among dietary treatments; Linear, linear contrast of dietary mixed selenium supplementation levels; Quadratic, quadratic contrast of dietary mixed selenium supplementation levels.

**Table 4:** Effects of dietary mixed selenium on immune responses of weaned pigs^1^.

	Dietary treatments				*p*-value		
Item^2^	CON	MSe3	MSe5	SEM	Diet	Linear	Quadratic
Cortisol, ng/mL							
Day 7	65.07	87.52	76.88	16.74	0.621	0.530	0.456
Day 14	102.06	88.14	88.29	20.08	0.855	0.615	0.822
Tumor necrosis factor-α, pg/mL							
Day 7	149.38	164.55	141.30	20.59	0.725	0.853	0.443
Day 14	152.29	132.57	161.01	22.87	0.673	0.868	0.390
Transforming growth factor-β1, pg/mL							
Day 7	2322.14	2206.00	2170.34	88.36	0.444	0.215	0.807
Day 14	2012.30	2359.68	2354.88	180.01	0.287	0.146	0.459
Interleukin-1β, pg/mL							
Day 7	78.31	78.43	68.51	9.31	0.656	0.463	0.599
Day 14	69.15	72.20	63.50	8.70	0.776	0.700	0.557
Interleukin-6, pg/mL							
Day 7	134.11^b^	151.49^a^	150.98^a^	4.39	0.021	0.011	0.200
Day 14	150.38	150.67	154.05	4.03	0.777	0.555	0.706

^1^Each value is the mean of 6 replicates (1 pig per pen).

^2^CON, a non-selenium-fortified diet based on corn and soybean meal; MSe3, CON + 0.15 ppm inorganic selenium + 0.15 ppm organic selenium; MSe5, CON + 0.25 ppm inorganic selenium + 0.25 ppm organic selenium; Diet, statistical comparisons among dietary treatments; Linear, linear contrast of dietary mixed selenium supplementation levels; Quadratic, quadratic contrast of dietary mixed selenium supplementation levels.

^a,b^Means with different superscript letters within each variable are different (*p* < 0.05).

**Table 5:** Effects of dietary mixed selenium on blood biochemical parameters of weaned pigs^1^.

	Dietary treatments				*p*-value		
Item^2^	CON	MSe3	MSe5	SEM	Diet	Linear	Quadratic
Total protein, g/dL	6.69^b^	7.30^ac^	6.88^bc^	0.16	0.049	0.276	0.026
Calcium, mg/dL	10.18	10.33	9.97	0.18	0.378	0.478	0.232
Inorganic phosphorus, mg/dL	8.09	8.13	8.23	0.31	0.952	0.775	0.905
Magnesium, mg/dL	2.44	2.52	2.18	0.13	0.182	0.219	0.161
Total cholesterol, mg/dL	92.33	102.83	92.75	6.83	0.486	0.857	0.243
Triglyceride, mg/dL	56.50	55.92	48.92	7.53	0.735	0.515	0.675
Glucose, mg/dL	91.92	98.92	108.25	9.06	0.460	0.229	0.806
Albumin, g/dL	4.07	4.18	4.07	0.11	0.672	0.919	0.384
Creatinine, mg/dL	1.15	1.00	0.90	0.15	0.527	0.266	0.999
Blood urea nitrogen, mg/dL	35.04	23.06	17.38	5.41	0.094	0.033	0.838
Aspartate aminotransferase, IU/L	40.33	35.83	61.67	12.43	0.320	0.292	0.277
Alanine aminotransferase, IU/L	49.50	43.58	41.92	3.39	0.281	0.124	0.748
AST:ALT, %	0.84	0.81	1.42	0.23	0.147	0.122	0.214

^1^Each value is the mean of 6 replicates (1 pig per pen).

^2^CON, a non-selenium-fortified diet based on corn and soybean meal; MSe3, CON + 0.15 ppm inorganic selenium + 0.15 ppm organic selenium; MSe5, CON + 0.25 ppm inorganic selenium + 0.25 ppm organic selenium; Diet, statistical comparisons among dietary treatments; Linear, linear contrast of dietary mixed selenium supplementation levels; Quadratic, quadratic contrast of dietary mixed selenium supplementation levels; AST:ALT, aspartate aminotransferase to alanine aminotransferase ratio.

^a–c^Means with different superscript letters within each variable are different (*p* < 0.05).

### Fecal microbial diversities and taxonomic relative abundance

**Figure 1:**
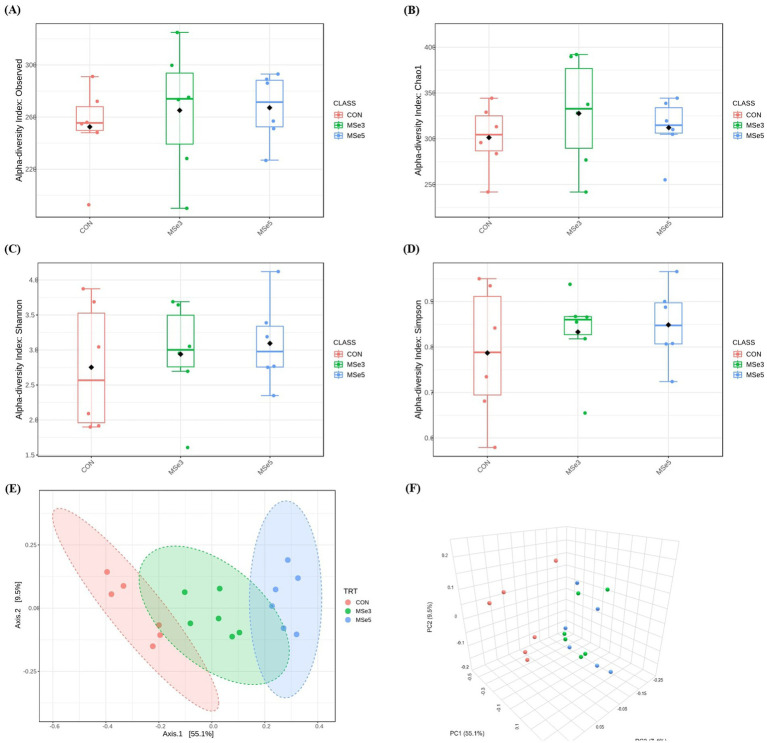
Effects of dietary mixed selenium on microbial alpha and beta diversity indices of weaned pigs. **(A)** Number of OTUs (*p* = 0.641), **(B)** Chao1 index (*p* = 0.717), **(C)** Shannon index (*p* = 0.810), **(D)** Simpson index (*p* = 0.796), and **(E,F)** PCoA plots based on the Bray–Curtis dissimilarity (*r*^2^ = 0.56, *p* = 0.001). CON, a non-selenium-fortified diet based on corn and soybean meal; MSe3, CON + 0.15 ppm inorganic selenium + 0.15 ppm organic selenium; MSe5, CON + 0.25 ppm inorganic selenium + 0.25 ppm organic selenium.

A total of 2,200,279 read counts were obtained from the feces of weaned pigs through 16S rRNA sequencing, with average reads of 122,238 ± 10,923 per sample. After quality filtering, the total number of read counts obtained was 220,982, with the average reads per sample being 12,277 ± 3,073. A summary of fecal microbial diversities in weaned pigs among dietary treatments is shown in Figure 1. There were no differences in microbial alpha diversity indices among the CON, MSe3, and MSe5 groups (Figures 1A–D: number of operational taxonomic units, Chao1, Shannon, and Simpson, respectively). Beta diversity based on the Bray–Curtis distance using PCoA plots among dietary treatments is illustrated in Figures 1E,F (2D and 3D plots, respectively). There was a difference (*r*^2^ = 0.56, *p* = 0.001) in the clustering of the fecal microbial community among the CON, MSe3, and MSe5 groups according to the level of dietary MSe supplementation (CON vs. MSe3, *r*^2^ = 0.37, *p* = 0.002; CON vs. MSe5, *r*^2^ = 0.62, *p* = 0.003; MSe3 vs. MSe5, *r*^2^ = 0.37, *p* = 0.004).

**Figure 2:**
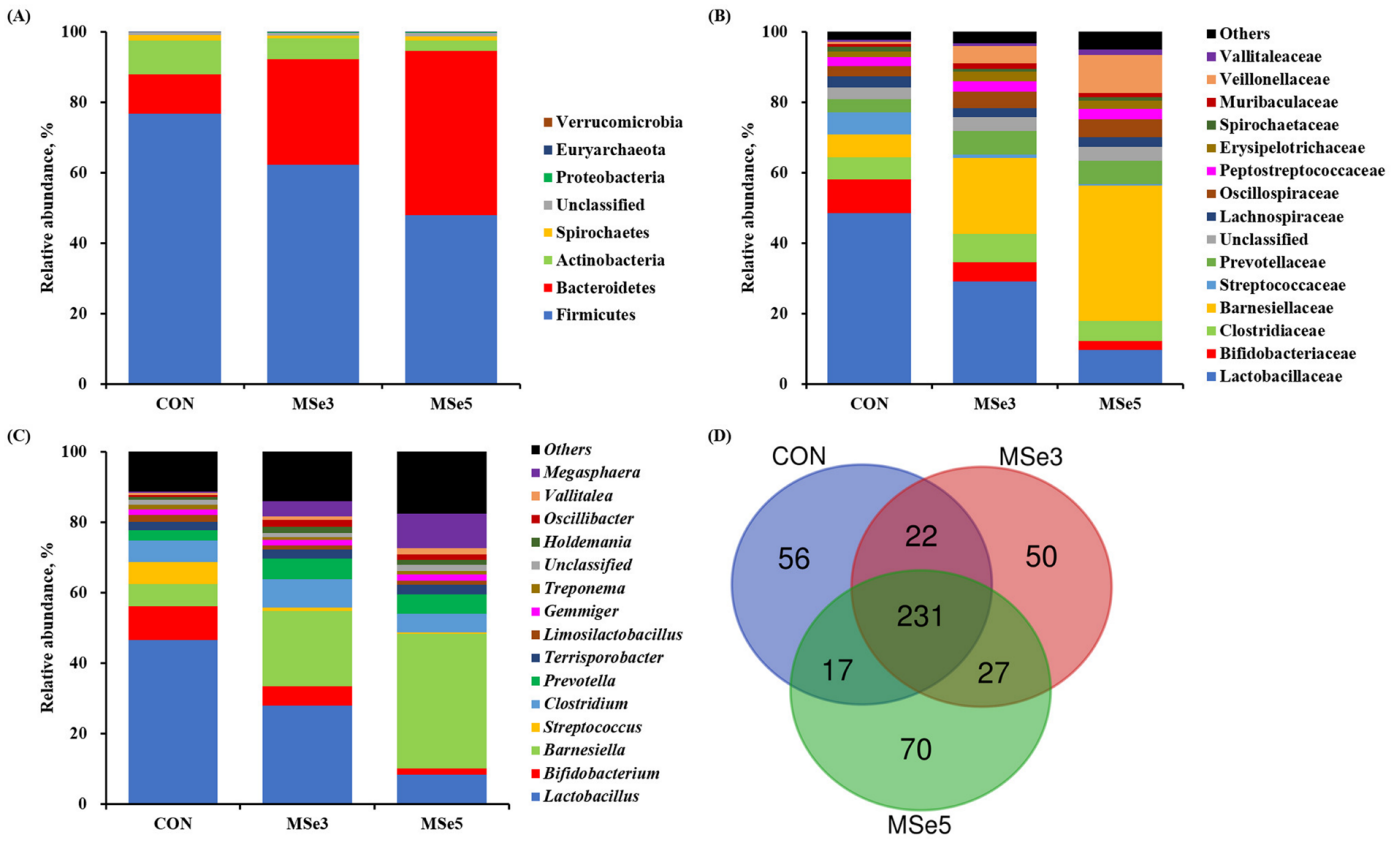
Effects of dietary mixed selenium on microbial taxonomic relative abundance in weaned pigs. The fecal microbiota of weaned pigs at the **(A)** phylum, **(B)** family, and **(C)** genus levels, respectively. The top 15 bacteria are presented at both family and genus levels, with the rest being included in others. **(D)** Venn diagram showing the unique and shared species among dietary treatments. CON, a non-selenium-fortified diet based on corn and soybean meal; MSe3, CON + 0.15 ppm inorganic selenium + 0.15 ppm organic selenium; MSe5, CON + 0.25 ppm inorganic selenium + 0.25 ppm organic selenium.

Microbial taxonomic profiling among dietary treatments is presented in Figure 2. At the phylum level, Firmicutes and Bacteroidetes were collectively accounted for 87.96–94.48% of the total sequences among dietary treatments (Figure 2A). A total of eight phyla were identified at the phylum level, and the top five most abundant phyla were Firmicutes (47.89–76.73%), Bacteroidetes (11.23–46.59%), Actinobacteria (3.10–9.63%), Spirochaetes (0.79–1.40%), and Proteobacteria (0.14–0.30%). At the family level, the top five most abundant families were Lactobacillaceae (9.72–48.50%), Barnesiellaceae (6.40–38.31%), Clostridiaceae (5.77–8.16%), Bifidobacteriaceae (2.53–9.52%), and Prevotellaceae (3.70–6.67%) regardless of dietary treatments (Figure 2B). At the genus level, the top five most abundant genera were *Lactobacillus* (8.40–46.59%), *Barnesiella* (6.40–38.31%), *Clostridium* (5.31–7.86%), *Bifidobacterium* (1.63–9.50%), and *Megasphaera* (0.43–9.85%) across dietary treatments (Figure 2C). According to the species-level Venn diagram, a total of 231 species overlapped among dietary treatments (Figure 2D). Additionally, 56, 50, and 70 species were unique to the CON, MSe3, and MSe5 groups, respectively.

**Figure 3:**
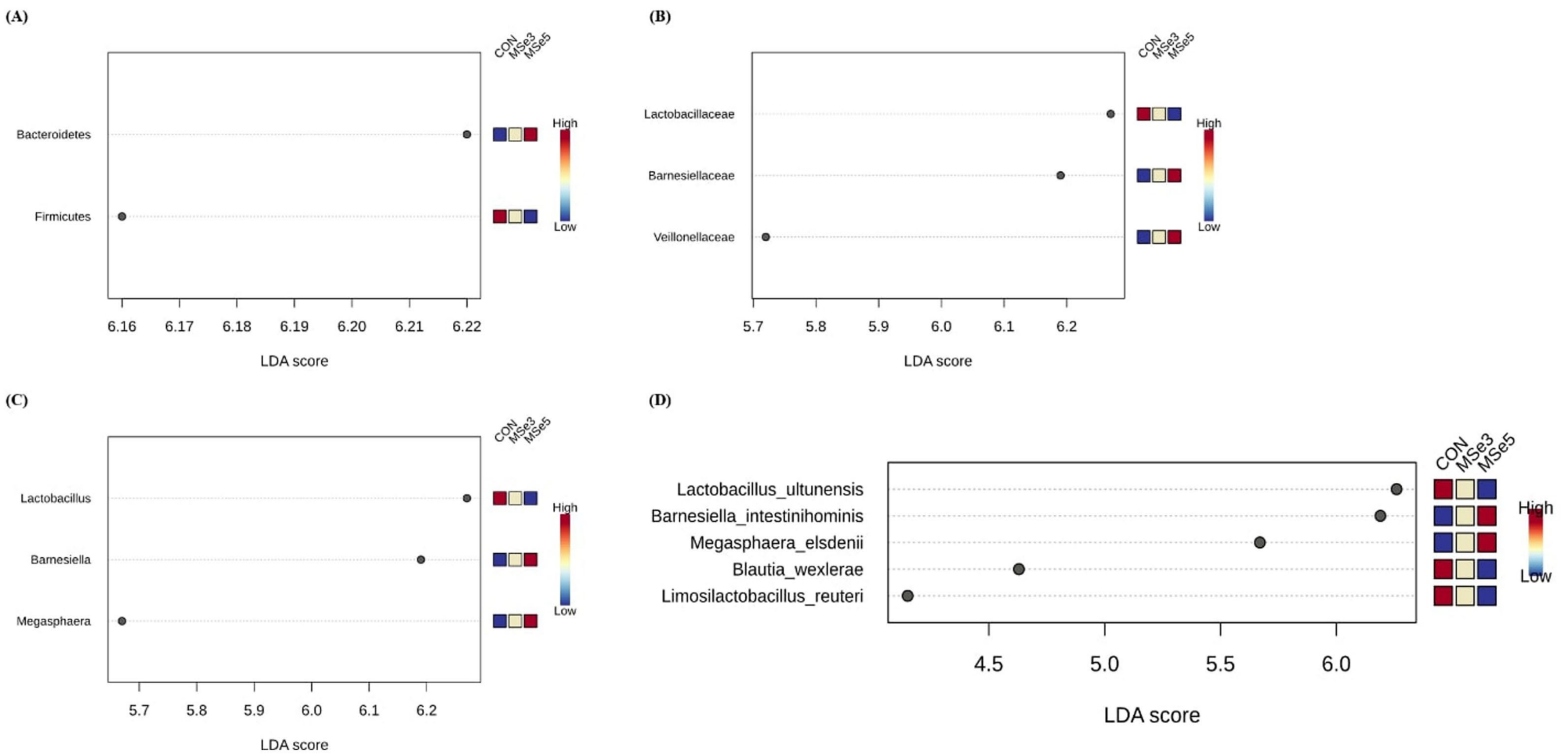
Effects of dietary mixed selenium on taxonomic features of weaned pigs determined by LEfSe analysis (LDA score > 2.0, *p* < 0.05). **(A)** Different phyla, **(B)** families, **(C)** genera, and **(D)** species among dietary treatments. CON, a non-selenium-fortified diet based on corn and soybean meal; MSe3, CON + 0.15 ppm inorganic selenium + 0.15 ppm organic selenium; MSe5, CON + 0.25 ppm inorganic selenium + 0.25 ppm organic selenium.

We determined which bacteria increased in abundance among the dietary treatments at the phylum, family, genus, and species levels based on the LEfSe plots (LDA ≥ 2.0; FDR adjusted *p* < 0.05) (Figure 3). The CON group had higher bacterial abundance of phylum Firmicutes (LDA = 6.17, FDR adjusted *p* = 0.008), family Lactobacillaceae (LDA = 6.27, FDR adjusted *p* = 0.006), genus *Lactobacillus* (LDA = 6.27, FDR adjusted *p* = 0.023), and species *Lactobacillus ultunensis* (LDA = 6.26, FDR adjusted *p* = 0.019), *Blautia wexlerae* (LDA = 4.63, FDR adjusted *p* = 0.016), and *Limosilactobacillus reuteri* (LDA = 4.15, FDR adjusted *p* = 0.016) than the MSe3 and Mse5 groups. In contrast, the MSe5 group had a higher bacterial abundance of the phylum Bacteroidetes (LDA = 6.22, FDR adjusted *p* = 0.004), families Barnesiellaceae (LDA = 6.19, FDR adjusted *p* = 0.006) and Veillonellaceae (LDA = 5.72, FDR adjusted *p* = 0.006), genera *Barnesiella* (LDA = 6.19, FDR adjusted *p* = 0.023) and *Megasphaera* (LDA = 5.67, FDR adjusted *p* = 0.023), and species *Barnesiella intestinihominis* (LDA = 6.19, FDR adjusted *p* = 0.016) and *Megasphaera elsdenii* (LDA = 5.67, FDR adjusted *p* = 0.016) than the CON and MSe3 groups.

### Correlation between featured microbial species and blood biochemical parameters

As presented in Table 6, a positive correlation (*r* = 0.60, *p* = 0.008) was determined between *Blautia wexlerae* and the BUN level. In contrast, a negative correlation (*r* = −0.49, *p* = 0.037) was observed between *Megasphaera elsdenii* and the BUN level.

**Table 6:** Pearson correlations between featured microbial species and serum biochemical parameters of weaned pigs^1^.

Item^2^	*Lactobacillus ultunensis*	*Barnesiella intestinihominis*	*Megasphaera elsdenii*	*Blautia wexlerae*	*Limosilactobacillus reuteri*
Calcium, mg/dL	*r* = 0.40, *p* = 0.098				
Magnesium, mg/dL	*r* = 0.41, *p* = 0.091				
Glucose, mg/dL			*r* = 0.45, *p* = 0.058		
Blood urea nitrogen, mg/dL		*r* = −0.45, *p* = 0.061	*r* = −0.49, *p* = 0.037	*r* = 0.60, *p* = 0.008	*r* = 0.42, *p* = 0.086
Alanine aminotransferase, IU/L	*r* = 0.46, *p* = 0.057				
AST:ALT, %	*r* = −0.41, *p* = 0.087		*r* = 0.43, *p* = 0.074		*r* = −0.43, *p* = 0.087

^1^Pearson correlation coefficients (*r*) with *p* < 0.10 are presented.

^2^CON, a non-selenium-fortified diet based on corn and soybean meal; MSe3, CON + 0.15 ppm inorganic selenium + 0.15 ppm organic selenium; MSe5, CON + 0.25 ppm inorganic selenium + 0.25 ppm organic selenium; AST:ALT, aspartate aminotransferase to alanine aminotransferase ratio.

## Discussion

This study determined that different levels of dietary MSe improved Se concentration in blood and modulated blood composition, inflammatory response, biochemical indices, and fecal microbiota in weaned pigs. However, dietary MSe addition did not affect the growth performance and post-weaning diarrhea frequency. In previous studies, the growth performance of weaned pigs was inconsistent depending on the source (dl-selenomethionine, sodium selenite, Se yeast, or hydroxy-selenomethionine) and level (0.1, 0.3, 0.375, 0.5, or 0.7 mg/kg of Se) of dietary Se (9, 27–29), but Se affected the health of pigs through its antioxidant or immunomodulatory effects against oxidative stress due to weaning. In addition, supplementation of dietary Se and Se-enriched products (0.3 mg/kg of Se nanoparticles; 0.3 ppm of Se mushroom powder; 0.3 mg/kg of OSe mushroom powder) alleviated diarrhea in early-weaned pigs by modulating antioxidant capacity and immunity (8, 17, 30). However, differences in results among studies may be attributed to the differences in the products and levels of dietary Se, as well as breeds, managing environment, or health conditions of pigs. Additionally, the lack of notable growth performance effects in our study may be due to the competitive utilization of the host and gut microbiota for nutritional MSe and the fact that MSe primarily contributed to systemic health indicators. This study confirmed that dietary MSe addition improved serum Se concentration. The results determined that blood Se levels in pigs were neither deficient nor toxic as indicated by reference values for Se status (29). However, the serum Se level in the CON group was close to the marginal level for livestock (0.05–0.08 ppm), suggesting that additional dietary Se supplementation should be considered. Clinical signs of Se deficiency, which are commonly observed after weaning (31, 32), are due to decreased blood nutritional indicators such as vitamin E and glutathione peroxidase with antioxidant properties (33–35) and increased oxidative stress at this time (36, 37). Thus, our findings indicated that MSe supplementation did not affect the growth performance and diarrhea frequency in weaned pigs but can lead to adequate Se status by improving the Se concentration in the blood.

Blood parameters reflect the physiological and health status of the animals and are influenced by nutritional status. In addition to its antioxidant properties, nutritional Se has anti-inflammatory properties that regulate the secretion of inflammatory cytokines, such as tumor necrosis factor-α and interleukins, by inhibiting nuclear factor-κB (NF-κB) activation through the regulation of SeP expression (38). In the present study, the serum interleukin-6 level was elevated in the early post-weaning period following dietary MSe addition, but it did not differ across the addition levels. Additionally, a quadratic tendency was observed on the number of white blood cells at the end of the experiment depending on the level of dietary MSe supplementation. These results indicate that dietary MSe modulated the systemic immune responses of weaned pigs. Consistent with our findings, it has been reported that dietary Se regulated cell-mediated immunity by improving serum interleukin levels under oxidative stress conditions such as weaning stress and bacterial challenge (27). In addition, adequate Se intake in a mice model resulted in increased serum IL-6 along with increased stress-related SeP expression (39). The cytokine IL-6 is known to have both pro- and anti-inflammatory properties, playing a crucial role in the immune system through macrophage activation (40). Meanwhile, Se exhibits anti-inflammatory properties by attenuating pro-inflammatory gene expression in macrophages (41). Therefore, as the immune system is immature in the post-weaning period due to impaired immune cell proliferation and increased oxidative stress (36, 42), MSe-triggered cytokine may effectively address diseases or infections during the stress period of early weaning. In addition, dietary MSe may be involved in the inflammatory response through regulated activation rather than inhibition of NF-κB, which plays an important role in macrophage activation. Furthermore, when comparing dietary MSe3 and MSe5, there were no differences in serum Se concentration and regulated cytokine level, suggesting that supplemental MSe3 may be more efficient in regulating systemic Se status and inflammatory cytokine.

In this study, we confirmed linear or quadratic effects of dietary MSe supplementation levels on hemoglobin and indices (i.e., hematocrit, MCH, and MCHC), reflecting the relationship between red blood cells and hemoglobin in the blood. Se deficiency affects the proportion of immature erythrocytes and hemoglobin in pigs (43). Moreover, Se and SePs positively influence not only protein oxidation but also erythrocyte and hemoglobin development against ROS generated under stress conditions (44). This is because erythrocytes, which constitute the main component of blood, are more susceptible to peroxidative damage due to their oxygen-carrying function and higher concentration. Interestingly, the hematological indices related to iron status showed an opposite trend with blood Se concentration. These results suggest that increased levels of dietary MSe supplementation may interfere with age-related erythropoiesis and/or development. Additionally, increased IL-6 can affect erythropoiesis by inducing a decrease in circulating iron associated with hepcidin activity, a hormone that regulates iron homeostasis (45, 46). However, this case is mainly associated with clinical anemia. Overall, the hemoglobin levels in all dietary treatments collected during the experimental period were not at the level of anemia (8.0 g/dL, borderline anemia; 7.0 g/dL or less, anemia) (47), and the Se concentration in the blood was not at the deficiency level as mentioned above. No hematological changes were observed in the absence of clinical signs of Se deficiency, despite the addition of various Se sources (48). Even the supplementation of toxic levels of dietary Se did not affect the hemoglobin and hematocrit levels of pigs (1). Thus, dietary Se may have more pronounced effects in improving erythrocytes, hemoglobin, or related indices under pathological conditions such as Se deficiency or anemia. However, since reductions in blood iron levels and changes in iron metabolism following Se supplementation have been reported in animal models (49, 50), further studies on the interaction between dietary MSe, hematological results, and iron metabolism are considered necessary.

This study revealed that dietary MSe altered total protein and BUN levels, which are used as indicators of protein metabolism and functions of the liver or kidneys. A greater total protein level may indicate increased protein synthesis in the body. Efficient utilization of protein means less conversion of excess nitrogen to urea for excretion. BUN reflects nitrogen utilization, which is derived from protein metabolism and finally excreted in the urine. In addition, BUN can indicate an efficient utilization of nutrients in the feed, which is related to increased feed efficiency (51). Furthermore, the reduction of BUN may alleviate metabolic stress in the tissues, indicating a healthier metabolic state. Previous studies have shown that Se deficiency increases protein metabolic end products (52), while Se supplementation alleviates them (53), which is consistent with our results. Taken together, dietary MSe supplementation indicates improved metabolic functions in the liver or kidneys, which are key Se metabolic organs, likely due to the antioxidant effects of MSe. In addition, an increased total protein level in the blood suggests that the effective action of immune-related proteins may be linked to the immunomodulatory effects of MSe. Thus, this study indicates the potential effects of different levels of dietary MSe on improving protein metabolism and supporting metabolic health in weaned pigs.

A variety of gut microbiota utilize Se for the expression of their own SePs, which may result in the competitive use of nutritional Se with the host (54). Additionally, through the animal model experiments, potential effects have suggested that the addition of dietary Se modulates gut microbial ecosystems and consequently enhances gut barrier functions as well as the SeP and antioxidant capacity (8, 55–57). However, the results on gut microbial diversities are inconsistent. In this study, there was no difference in alpha diversity of fecal microbiota, but different clusters were identified according to dietary MSe supplementation in beta diversity. These results may be due to the relatively minor effects of MSe on the majority of the microbiota of weaned pigs. On the other hand, the MSe sensitivity of specific microbial communities or low abundance communities may have resulted in their growth or inhibition. Consequently, dietary MSe did not alter the richness and evenness of the fecal microbiota of weaned pigs, but the overall composition was dissimilar. Therefore, to evaluate gut health, which is complex and has diverse function interactions, not only diversities but also the taxonomic abundance of each microbiota that constitutes the gut microbial communities should be considered.

We identified the microbiota that can be characterized as regulated by dietary MSe down to the species level using LEfSe analysis. Increasing levels of dietary MSe elevated the abundance of genera *Barnesiella* and *Megasphaera* in the gut of weaned pigs, while decreasing the abundance of genus *Lactobacillus*. Additionally, the MSe5 group was characterized by the species *Barnesiella intestinihominis* and *Megasphaera elsdenii* included in the increased genera, whereas the non-MSe group was characterized by the species *Lactobacillus ultunensis*, *Blautia wexlerae*, and *Limosilactobacillus reuteri*. Genus *Barnesiella* and species *B. intestinihominis* regulate microbial composition by restricting the colonization of pathogenic antibiotic-resistant bacteria and improve anticancer effects by stimulating immunomodulation (58, 59). In addition, the genus *Megasphaera* and species *M*. *elsdenii* utilize intestinal lactic acid in pigs to produce short-chain fatty acids, which are used as energy sources for the host and play important roles in intestinal health (60). Moreover, antibiotic-sensitive *M. elsdenii* has a potential probiotic approach that delays the dominance of antibiotic-resistant strains (61). Meanwhile, contrary to our results, when pigs were fed OSe-enriched products or OSe diets, the abundance of *Lactobacillus* increased (16, 17, 30) or *Megasphaera* decreased (57), respectively. Resulting differences may be related to the sources and levels of dietary Se as well as biological and environmental factors in the experiment. Collectively, this study could not clearly determine the individual effect of dietary ISe or OSe on altering the fecal microbiota of weaned pigs, but the relative dominance of *Barnesiella* spp. (*B. intestinihominis*) and *Megasphaera* spp. (*M. elsdenii*) in weaned pigs fed dietary MSe may suggest an interaction on the nutritional utilization of Se in the gut. Interestingly, Se has potential antimicrobial effects and, in particular, it was reported that biogenic Se nanoparticles exhibited antibacterial activity against drug-resistant bacteria and had potential as antibacterial agents (4, 62, 63). Based on the characterized microbiota following MSe addition, it was hypothesized that these bacteria may be less sensitive to the antibacterial activity of MSe or may have been metabolically utilized to stimulate the antibacterial activity. Moreover, these species were negatively correlated with the BUN of weaned pigs. The increase in microbial species directly or indirectly induced by dietary MSe may have preferentially altered the gut microbial environment and then influenced the nutrient metabolic efficiency of the host tissues. In contrast, the species *Blautia wexlerae* identified in non-Se pigs showed a positive correlation with BUN, indicating the potential for modulation of gut health and systemic nutrient utilization in weaned pigs by dietary MSe addition. Furthermore, gut-mediated signals such as microbial metabolites are important for gut and host health. Thus, the metabolites or functions of the correlated microbiota may have influenced the health of the host by interacting with the gut–organ (e.g., liver and kidney) axis. Further studies evaluating the metabolic and functional profiles of gut microbiota following dietary MSe supplementation would be helpful in establishing the effects of dietary MSe in pigs.

## Conclusion

In conclusion, the present study demonstrated that supplementation of dietary MSe to the corn and soybean meal-based non-Se-fortified diet enhanced serum Se concentration, modulated systemic health parameters, and modified fecal microbiota in weaned pigs. However, the MSe was not effective on growth performance and frequency of post-weaning diarrhea. Supplemental MSe contributed to the contrast effects of different addition levels on hematological indices, increased cytokine level, and improved nutritional metabolic indices following enhanced systemic Se level in weaned pigs. In addition, gut microbiota shifted by supplemental MSe correlated with improved blood biochemical index, suggesting the modulatory effects of dietary MSe on gut microbiota and host health. Overall, dietary MSe indicated potential immune and gut microbiota modulatory effects. However, excessive supplementation of Se can cause adverse effects such as toxicity. Similarly, an overdose of dietary MSe exceeding nutrient requirements may result in an imbalance of systemic responses and gut microbiota. Thus, exploring the interaction of gut microbiota with metabolic functional profiles in future studies would be beneficial to understand the potential effects of MSe. Moreover, considering additional approaches to SeP and antioxidant markers could efficiently reveal the MSe effects. Our results may provide a novel approach to the health of weaned pigs following dietary MSe addition.

## Data Availability

The datasets presented in this study can be found in online repositories. The names of the repository/repositories and accession number(s) can be found: https://www.ncbi.nlm.nih.gov/, PRJNA1222547.

## Footnotes

^1^ https://www.microbiomeanalyst.ca/
